# Supplementary material for: Roles of beta synchronization for motor skill acquisition change after stroke
Source: Brain Commun. 2026 Mar 11;8(2):fcag077. doi: 10.1093/braincomms/fcag077 (PMC13010071; doi:10.1093/braincomms/fcag077)
Supplement: fcag077_Supplementary_Data [file fcag077_supplementary_data.pdf]

Supplementary Material to "Roles of beta synchronisation for motor skill acquisition change after stroke"

Lena S. Timmsen, Benjamin Haverland, Silke Wolf, Charlotte J. Stagg, Jan Feldheim, The Vinh Luu, Robert Schulz, Till R. Schneider, Fanny Quandt* & Bettina C. Schwab*

*shared senior authorship

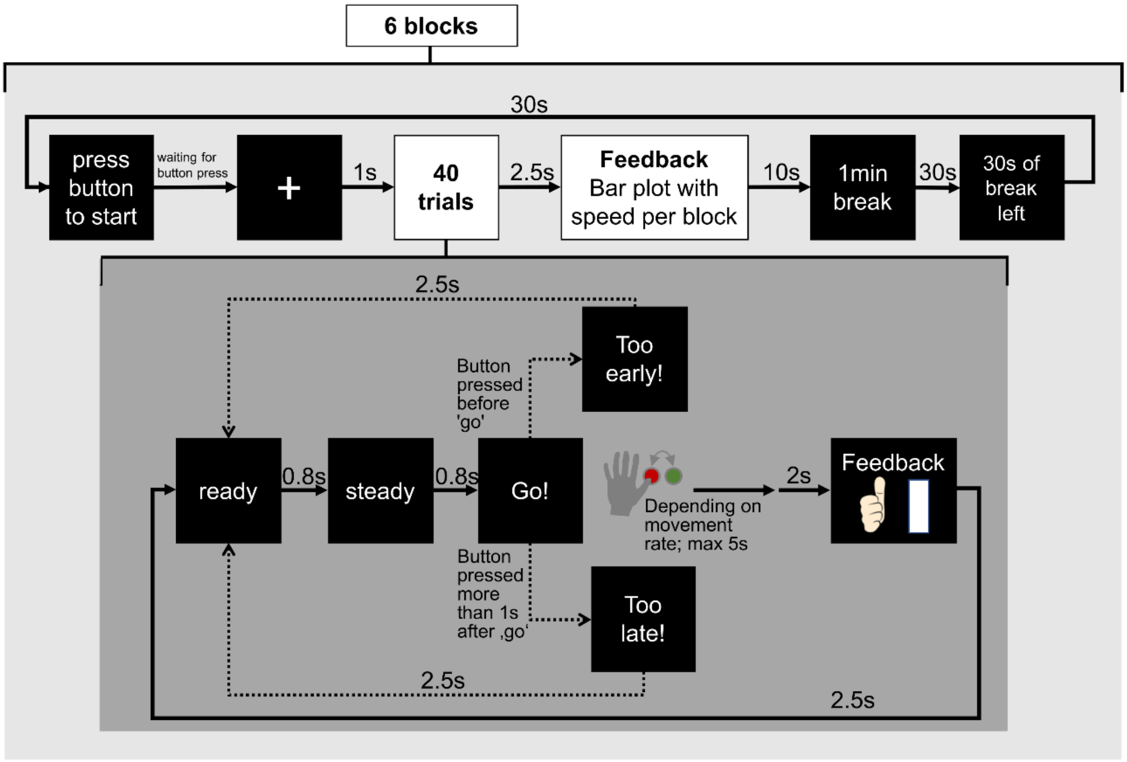

Supplementary Figure 1. Task design and stimulus timing. The task consisted of one baseline block of 20 trials and six measurement blocks, each containing 40 trials. During each trial, participants performed four alternating button presses in maximum speed in response to a visual cue. Participants received real-time feedback on their *movement rate* after each trial. The feedback consisted of a thumb which was presented up, middle or downwards indicating whether the just performed trial was faster, slower or similar in comparison to the average of all trials from all blocks performed before. Next to the thumb a bar representing the absolute speed from the just performed trial was shown. Additional feedback showed the block-averaged *movement rate* for the preceding block after each block. Between blocks, participants were given a one-minute rest period. If the button presses were executed too early or too late it was proceeded with the next trial. Black areas represent text shown on screen during the task. The arrows with time information indicate how long a screen was presented.

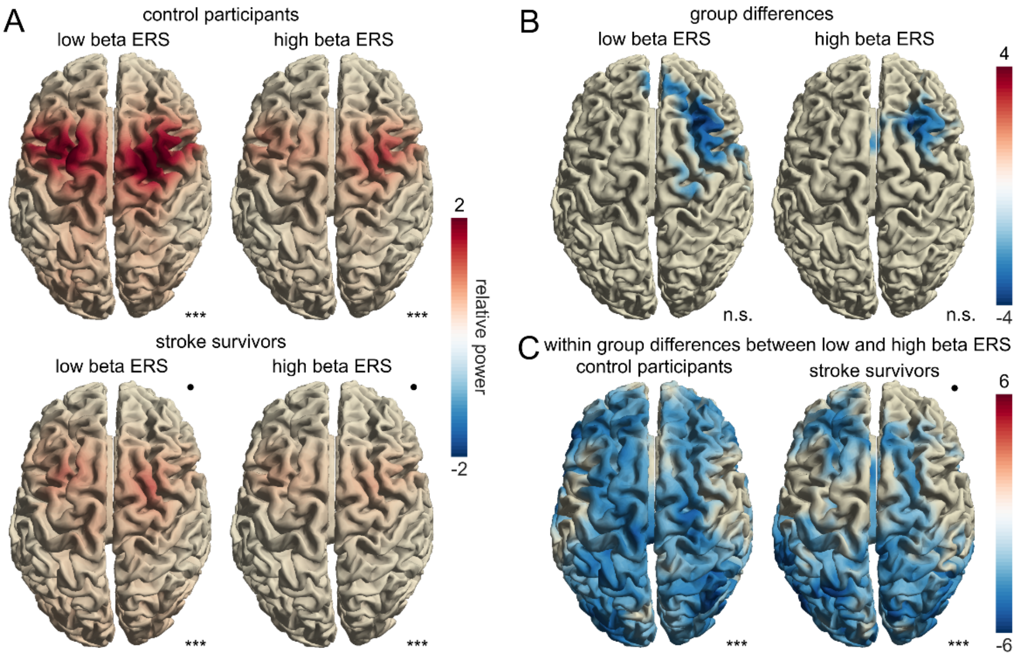

Supplementary Figure 2. Low beta ERS power is stronger than high beta ERS power in both groups. (**A**) Beta ERS in the low beta band (14 – 20 Hz) and high beta band (21 – 29 Hz) for each group. Permutation-based cluster statistics revealed significant low as well as high beta ERS in both groups compared to baseline (control participants, n=15: low beta ERS: $p < 0.001$, high beta ERS: $p < 0.001$, stroke survivors, n=14: low beta ERS: $p < 0.0001$, high beta ERS: $p < 0.0001$, not corrected for multiple comparisons). (**B**) Results of the permutation-based cluster statistics comparing low and high beta ERS between groups. Non-significant clusters indicated a trend toward reduced low and high beta ERS in stroke survivors compared to control participants (low beta ERS: $p = 0.13$, high beta ERS: $p = 0.15$, not corrected for multiple comparisons; stroke survivors n=15, control participants n=14). (**C**) Low beta ERS power was significantly stronger than high beta ERS power in both groups, as revealed by permutation-based cluster statistics (control participants, n=15: $p_{cor} < 0.001$, stroke survivors, n=14: $p_{cor} < 0.001$, corrected for two comparisons). The black dot represents the lesion side (right hemisphere). Significance markers: *n.s.* = not significant, ***$p < 0.001$.

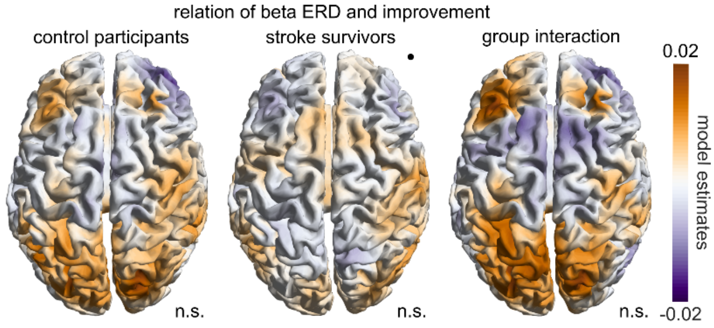

Supplementary Figure 3. Beta ERD was not significantly linked to *improvement*. Model estimates for the factor *improvement* (model: *beta ERD ~ performed hand + movement rate block 1 + improvement),* calculated with permutation-based cluster statistic for each group, as well as the model estimates for the *improvement* x group interaction (model: *beta ERD ~ performed hand + movement rate block 1 + improvement x group)*. No significant clusters were found (control participants, n=15: $p = 0.44$, stroke survivors, n=14: $p = 0.39$, interaction model: no cluster found, not corrected for multiple comparisons). The black dot represents the lesion side (right hemisphere). Significance markers: *n.s.* = not significant.

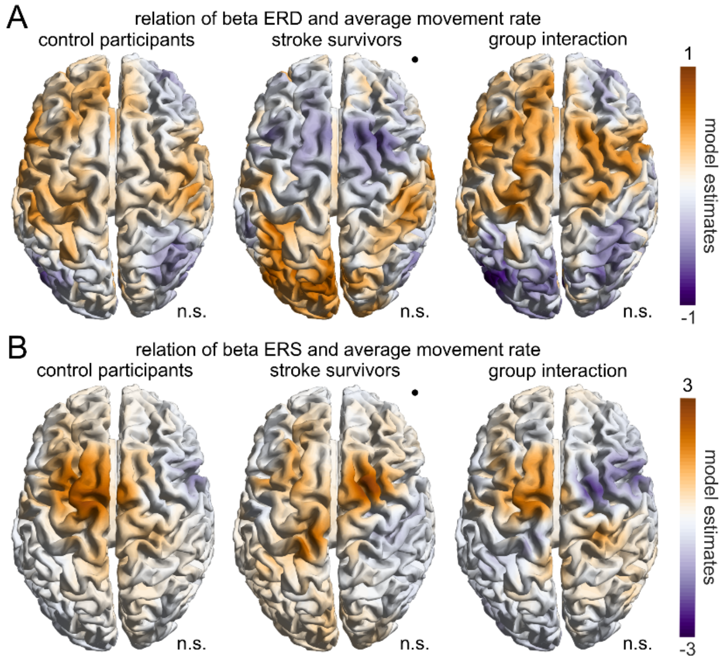

Supplementary Figure 4. Beta ERD and beta ERS were not significantly related to *movement rate*. (**A**) Model estimates for factor average *movement rate* (model: *beta ERD ~ performed hand + average movement rate),* calculated with permutation-based cluster statistic for each group, as well as the model estimates of the interaction *movement rate* x group (model *beta ERD ~ performed hand + average movement rate x group)*. No significant clusters were found (control participants, n=15: p = 0.44, stroke survivors, n=14: p = 0.29, interaction model: 0.46, not corrected for multiple comparisons). (B) Model estimates for the factor *movement rate* (model: *beta ERS ~ performed hand + average movement rate)* calculated with permutation-based cluster statistic for each group, as well as the model estimates of the interaction *movement rate* x group (model *beta ERS ~ performed hand + average movement rate x group)*. No significant clusters were found (control participants, n=15: p = 0.34, stroke survivors, n=14: p = 0.15, interaction model: 0.43, not corrected for multiple comparisons). The black dot represents the lesion side (right hemisphere). Significance markers: *n.s.* = not significant.

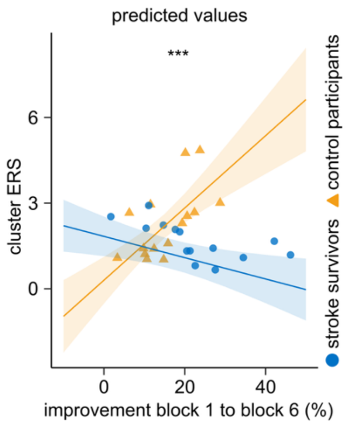

Supplementary Figure 5. Linear model showing the interaction of group x *block 1 to block 6 improvement* in relation to the cluster ERS, defined as the maximum individual beta ERS within the cluster. Model predictions (line) with shaded areas indicating the 95% confidence interval. Participant data points are shown as circles (stroke survivors n = 14) and triangles (control participants n = 15). Asterisks indicate significance of interaction term. Significance markers: ***p < 0.001.

Supplementary Table 1. Demographic and clinical data of control participants.

Control participant	Sex	Age	Performed hand	Fastest block in task	MRS	NIHSS	UEFM	ARAT	Grip force	KG force	NHPT	BBT
1	M	56	L	6	0	0	66	57	0.92	0.96	0.35	67
2	M	78	L	6	0	0	66	57	0.95	0.83	0.43	68
3	W	62	L	5	0	0	66	57	0.94	1.02	0.44	56
4	M	68	L	4	0	0	66	57	0.88	1.01	0.47	49
5	M	60	L	3	0	0	64	55	1.06	1.06	0.39	60
6	W	55	L	6	0	0	66	57	1.05	0.87	0.45	78
7	M	60	L	4	0	0	66	57	0.90	0.96	0.38	65
8	M	63	L	6	0	0	66	57	1.01	0.95	0.43	62
9	W	63	L	5	0	0	66	57	1.12	1.00	0.43	63
10	M	62	L	6	0	0	66	57	0.88	0.96	0.36	70
11	W	76	R	6	0	0	66	57	1.02	0.97	0.56	75
12	W	76	R	6	0	0	66	57	3.68	1.13	0.49	67
13	W	76	R	6	0	0	66	57	1.18	0.80	0.47	65
14	W	52	R	4	0	0	64	57	1.08	0.93	0.43	66
15	M	61	R	6	0	0	66	57	1.06	1.03	0.56	77
Mean control	F: 7	64.5		5.27	0	0	65.7	56.87	1.18	0.97	0.44	65.87
SD control		8.4		1.03	0	0	0.7	0.52	0.7	0.09	0.06	7.65
Mean stroke	F: 7	65.4		4.71	1.1	0.6	61.1***	54.93**	0.88	1.00	0.35*	49.14**
SD stroke		9.3		1.44	0.7	0.8	6.1	3.45	0.33	0.25	0.12	14.73

*$p < 0.05$, **$p < 0.01$, ***$p < 0.001$, statistical test of stroke vs control group, uncorrected. Values of grip force and key grip (KG) force are given as ratios of the affected to the non-affected hand in stroke survivors, and of the performing to the non-performing hand in control participants. Values of MRS (modified Rankin Scale), NIHSS (National Institutes of Health Stroke Scale), ARAT (Action Research Arm Test), NHPT (Nine Hole Peg Test, pegs/s) and BBT (Box and Blot Test, blocks/min) are reported for the affected hand in stroke survivors and performing hand for control participants. UEFM (Fugl-Meyer Assessment of the Upper Extremity) is stated as the absolute score for both groups. TAS: time after stroke, SD: standard deviation, TC: thalamocapsular, CR: corona radiata, CI: capsula interna, BG: basal ganglia, PLCI: posterior limb of the capsula interna, MED: media infarct, PO: pons, PRE: precentral gyrus

Supplementary Table 2. Individual performance data of all participants.

cotrol participant	movement rate (1/s)						fastest block	improvement (%)
	block 1	block 2	block 3	block 4	block 5	block 6		
1	1.255	1.356	1.498	1.453	1.529	1.616	6	28.74
2	1.065	0.955	1.087	1.137	1.144	1.173	6	10.16
3	1.103	1.109	1.179	1.190	1.330	1.275	5	20.59
4	1.018	1.089	1.107	1.135	1.128	1.118	4	11.52
5	1.394	1.432	1.567	1.513	1.554	1.516	3	12.41
6	1.215	1.326	1.300	1.300	1.356	1.408	6	15.87
7	1.346	1.453	1.531	1.545	1.467	1.490	4	14.79
8	1.345	1.521	1.612	1.602	1.545	1.646	6	22.41
9	1.296	1.344	1.377	1.376	1.433	1.432	5	10.62
10	1.074	1.123	1.193	1.238	1.232	1.282	6	19.37
11	1.086	1.134	1.201	1.266	1.223	1.304	6	20.15
12	1.260	1.184	1.124	1.151	1.253	1.302	6	3.32
13	1.014	1.051	0.999	1.076	1.063	1.077	6	6.28
14	1.011	1.025	1.071	1.110	1.104	1.069	4	9.81
15	1.160	1.316	1.380	1.381	1.373	1.436	6	23.75

stroke survivor	movement rate (1/s)						fastest block	improvement (%)
	block 1	block 2	block 3	block 4	block 5	block 6		
1	1.037	1.146	1.107	1.190	1.176	1.123	4	14.71
2	0.791	0.793	0.874	0.823	0.831	0.814	3	10.44
3	0.588	0.640	0.708	0.564	0.622	NaN	3	20.52
4	0.869	0.984	1.019	0.991	0.981	1.066	6	22.59
5	0.674	0.789	0.737	0.802	0.860	0.831	5	27.55
6	1.248	1.310	1.311	1.293	1.412	1.469	6	17.66
7	0.813	0.929	0.986	0.958	1.022	1.156	6	42.17
8	1.004	1.215	1.306	1.375	1.398	1.467	6	46.17
9	1.170	1.286	1.221	1.159	1.219	1.299	6	11.08
10	1.069	1.149	1.195	1.234	1.269	1.268	5	18.77
11	1.240	1.262	1.180	1.180	1.117	1.144	2	1.72
12	0.835	0.902	0.944	0.973	1.012	1.011	5	21.30
13	0.664	0.772	0.893	0.842	0.851	0.878	3	34.49
14	0.762	0.826	0.862	0.878	0.888	0.968	6	27.03

Supplementary Table 3. Results of linear-mixed effects model calculated with cluster-based permutation statistics on the whole brain MEG data. Not corrected for multiple comparisons (stroke survivors n=14, control participants n=15).

**group model**	control participants					stroke survivors				
		p	cluster statistics	SD	CI		*p*	cluster statistics	SD	CI
beta ERD ~ performed hand + movement rate block 1 + improvement	positive cluster	0.44	12.24	0.02	0.03	positive cluster	0.39	13.6	0.02	0.03
beta ERS ~ performed hand + movement rate block 1 + improvement	positive cluster	**<0.001**	5.3×10^3	<0.001	0.002	negative cluster	0.38	-29.64	0.02	0.03
						positive cluster	0.42	30.79	0.02	0.03
beta ERD ~ performed hand + average movement rate	positive cluster	0.44	11.02	0.02	0.03	positive cluster	0.29	23.67	0.01	0.03
beta ERS ~ performed hand + average movement rate	negative cluster	0.34	-6.86	0.02	0.03	positive cluster	0.15	118.97	0.01	0.02
	positive cluster	0.41	6.17	0.02	0.03					

**interaction model**					
		p	cluster statistics	SD	CI
beta ERD ~ performed hand + movement rate block 1 + improvement x group	no cluster found				
beta ERS ~ performed hand + movement rate block 1 + improvement x group	positive cluster	**0.002**	3.67×10^3	0.001	0.003
beta ERD ~ performed hand + average movement rate x group	negative cluster	0.46	-23.85	0.02	0.03
beta ERS ~ performed hand + average movement rate x group	negative cluster	0.58	-2.77	0.02	0.03
	positive cluster	0.43	8.22	0.02	0.03

Supplementary Table 4. Exploratory analysis reveals a significant interaction of BBT with group and improvement. The model "cluster ERS ~ performed hand + movement rate block 1 + improvement x group x parameter" was calculated for each parameter (Fugl-Meyer Assessment of the Upper Extremity (FMUE), Action Research Arm Test (ARAT), Box and Block Test (BBT), Nine Hole Peg Test (NHPT), grip force, key grip force, fractional anisotropy of the cortical spinal tract (CST FA ratio), age). The "cluster ERS" indicates the individual maximum beta ERS within the cluster in which we found a significant association between beta ERS and *improvement*. The standard deviations (SE) and p-values (p) are displayed for each predictor variable. Additionally, R-squared, F-statistics and p-values of each model are stated. All values are uncorrected for multiple comparisons (stroke survivors n=14, control participants n=15).

Parameter	FMUE		ARAT		BBT		NHPT		Grip force		Key grip		CST FA ratio		Age	
	SE	p	SE	p	SE	p	SE	p	SE	p	SE	p	SE	p	SE	p
Intercept	1.5E+02	6.4E-02	2.4E+01	9.8E-01	5.0E+00	**4.9E-03**	4.1E+00	1.9E-01	1.5E+00	2.7E-01	4.2E+00	1.4E-01	4.6E+00	4.6E-01	3.0E+00	9.4E-01
Performed hand r	2.8E-01	**1.2E-04**	3.0E-01	**6.3E-04**	2.8E-01	**7.1E-04**	2.9E-01	**3.5E-02**	3.4E-01	**2.7E-03**	2.8E-01	**1.5E-03**	4.0E-01	**1.1E-02**	2.6E-01	**1.1E-03**
Performance block 1	1.1E+00	**3.3E-02**	1.0E+00	3.3E-01	8.7E-01	4.7E-01	8.7E-01	9.9E-01	1.1E+00	3.8E-01	1.0E+00	3.0E-01	1.0E+00	2.6E-01	8.8E-01	3.3E-01
Improvement x Group stroke x Parameter	2.0E-01	7.81E-02	NA	NA	4.7E-03	**1.6E-02**	4.0E-01	7.1E-01	2.2E-01	5.5E-01	4.2E-01	1.4E-01	6.9E-01	7.2E-01	3.8E-03	2.6E-01
R squared	7.6E-01		7.0E-01		8.0E-01		8.2E-01		7.1E-01		7.3E-01		7.4E-01		7.9E-01	
F statistics	6.7E+00		5.9E+00		8.6E+00		9.3E+00		5.2E+00		5.8E+00		4.8E+00		7.8E+00	
Model p-value	**2.5E-04**		**6.1E-04**		**4.7E-05**		**2.6E-05**		**1.2E-03**		**6.1E-04**		**3.7E-03**		**9.1E-05**	
